# Association between inflammatory-response gene polymorphisms and risk of acute kidney injury in children

**DOI:** 10.1042/BSR20180537

**Published:** 2018-12-14

**Authors:** Jing He, Guoyan Xie, Hui Wu, Song Xu, Jun Xie, Youyuan Chen, Xinqian Zhao

**Affiliations:** 1 Department of Pediatrics, The First People’s Hospital of Bijie, Bijie 551700, Guizhou Province, China; 2 Department of Radiology, The First People’s Hospital of Bijie, Bijie 551700, Guizhou Province, China; 3 Department of Clinical Laboratory, The First People’s Hospital of Bijie, Bijie 551700, Guizhou Province, China

**Keywords:** Acute kidney injury (AKI), biomarkers, inflammation, pediatric, polymorphisms

## Abstract

In the present study, we investigated the association of 12 polymorphisms in six inflammatory-response genes (*TNF, IL6, IL10, IL18, NFKB1* and *NFKBIA*) with risk of acute kidney injury (AKI) in children. The polymorphisms were genotyped in 1138 children with AKI and 1382 non-AKI controls. Logistic regression analysis was performed to calculate the odds ratio for estimating the risk association. After accounting for Bonferroni correction and adjustment for potential confounders, significant association was observed for *NFKB1* rs28362491, *NFKBIA* rs2233406 and *NFKBIA* rs696 polymorphisms (*P* < 0.004). All three polymorphisms were associated with a reduced risk of AKI. For rs28362491 polymorphism, the OR for ID vs. II comparison was 0.75 (95% CI = 0.58–0.83) while that for DD vs. II was 0.44 (95% CI = 0.30–0.67). For rs2233406 polymorphism, the CT vs. CC comparison showed an OR of 0.90 (95% CI = 0.39–0.99), while the TT vs. CC comparison showed an OR of 0.43 (95% CI = 0.33–0.80). For rs696 polymorphism, the OR for AG vs. AA comparison was 0.71 (95% CI = 0.43–0.89), while the GG vs. AA comparison showed an OR of 0.39 (95% CI = 0.21–0.71). In conclusion, *NFKB1* rs28362491, *NFKBIA* rs2233406 and *NFKBIA* rs696 polymorphisms may serve as biomarkers for predicting risk of AKI in children.

## Introduction

Acute kidney injury (AKI) is a significant concern in intensive care units, as it is associated with a substantial burden of morbidity, mortality and expenditures in the healthcare sector [1]. AKI is characterized by a sudden and unexpected decline in renal function that occurs rapidly, usually as a complication of other medical conditions or procedures, such as septic shock, cardiac surgery and liver transplantation. However, the reason why only some of the patients with these medical conditions develop AKI remains incompletely explained. Among the adults, several clinical risk factors of AKI have been identified, including aortic arteriosclerosis, advancing age, hypertension and diabetes mellitus [2]. However, these risk factors are normally not applicable to the pediatric patients. Thus, there is a need for identification of additional factors that can be reliably used to predict whether an individual, especially a child, would develop the syndrome.

Genetic factors have been proposed to contribute to inter-individual differences in susceptibility to AKI [3,4]. A number of studies have been performed to investigate the relationship between various genetic polymorphisms and risk of AKI [3,4]. However, most of these studies were conducted on adult patients, but not among the pediatric population. Moreover, the majority of these studies employed relatively small sample sizes. In addition, the association between genetic polymorphisms and disease risk has been known to vary across populations, and very little (if any) studies have been performed in the Chinese population. This represents gaps in the literature that need to be addressed.

In recent years, inflammatory process has been pinpointed as a key player in the pathophysiology of AKI [5,6]. Infiltration of inflammatory cells has been observed in the injured kidney. It is thought that these cells play important roles in initiating and sustaining the kidney injury by releasing oxygen radicals and vasoconstrictors, as well as mediating the release of endothelin and inhibiting the release of nitric oxide, which can result in direct endothelial injury [6]. Given the importance of inflammatory process in the development of AKI, polymorphisms in inflammation-related genes may influence the susceptibility of an individual to AKI.

Among the important inflammation-related genes that could play a role in AKI are *IL6, IL10, NFBK1, NFKBIA, IL18* and *TNF. IL6* encodes for interleukin-6, which has been shown to induce a cytokine-dependent cell-mediated immune response which causes kidney damage [7]. In addition, plasma level of interleukin-6 has been found to serve as a good biomarker for predicting AKI [8]. Three promoter polymorphisms within the *IL6* genes, namely rs1800795, rs1800796 and rs1800797 polymorphisms, have been shown to influence the expression and secretion of the cytokine [9]. Thus, these polymorphisms serve as ideal candidates for genetic association studies in AKI. Several works have found that the minor allele of rs1800795 and rs1800797 are present at a low frequency in the general population. We did not exclude the two polymorphisms from the study because we hypothesize that these uncommon SNPs are either evolutionarily conserved or functionally important, thus their genetic variation could play a causative role in AKI [10]. Moreover, it has been demonstrated previously that even polymorphisms with very low minor allele frequencies could provide meaning information and potential utility as a biomarker, and should not be removed from the analysis [11].

*IL10* encodes for interleukin-10, whose plasma level has also been associated with AKI [8,12]. Interleukin is implicated in AKI pathogenesis due to its anti-inflammatory role. It is known that interleukin-10 facilitates the inhibition of immune cells and secretion of pro-inflammatory mediators, thus disrupting the repair process after kidney injury [12]. Promoter polymorphisms within the *IL10* gene have been shown, or proposed, to influence the level of the interleukin. These include the *IL10* rs1800896 and rs3021097 polymorphisms [13,14]. Examining the association between the polymorphisms and AKI risk could potentially provide important insights into their role as a biomarker.

*NFKB1* encodes for nuclear-factor kappa beta 1 (NF-κB1), which does not play a direct role in inflammation but serve as the central regulator of a huge array of molecules involved in the inflammatory process. Hence, it is not surprising that *NFKB1* and its related genes are commonly implicated in the pathogenesis of AKI [15,16]. An insertion–deletion polymorphism (rs28362491) within the promoter region of *NFKB1* gene could affect its level and functions, thus causing disruption to the inflammatory balance in the cells. As such, it is reasonable to hypothesize that the polymorphism could be associated with AKI risk. In addition, it is known that an optimal level of NF-κB1 is essential for its regulatory functions [17]. The cellular level of NF-κB1 is controlled tightly by IκBα, which is encoded by *NFKBIA* [17]. The rs2233406 and rs696 polymorphisms of the *NFKBIA* gene, which are respectively located at the promoter and 3′UTR region of the gene, could affect its expression. This can in turn, affect its inhibitory roles, leading to a disrupted nuclear-factor kappa beta pathway, which eventually causes AKI. Thus, there is a potential association between the *NFKBIA* polymorphisms and AKI risk.

Interleukin-18, encoded by *IL18*, is yet another cytokine that has been implicated in AKI. Animal studies [18] have demonstrated that the interleukin could induce acute tubular necrosis of the kidney. Studies in humans have also linked interleukin-18 to AKI [19]. Thus, a disrupted level of interleukin-18 could serve as a risk factor for AKI. Promoter polymorphisms in *IL18* gene may influence the level of the cytokine. Two such *IL18* polymorphisms are the rs1946518 and rs187238 polymorphisms. Therefore, there could be an association between the two polymorphisms with AKI risk.

Finally, tumor necrosis factor, encoded by *TNF*, is one of the most classic proinflammatory mediators. The cytokine has been linked to many kidney diseases, including AKI [20,21]. Two *TNF* promoter polymorphisms (rs1799964 and rs1800629) have been frequently implicated in the regulation of its transcriptional activity [22]. As such, we hypothesized that the polymorphisms could be associated with risk of AKI.

In this work, we aimed to examine the association of *IL6* rs1800795, *IL6* rs1800796, *IL6* rs1800797, *IL10* rs1800896, *IL10* rs3021097, *NFKB1* rs28362491, *NFKBIA* rs2233406, *NFKBIA* rs696, *IL18* rs1946518, *IL18* rs187238, *TNF* rs1799964 and *TNF* rs1800629 polymorphisms with AKI risk among the pediatric population in China.

## Materials and methods

### Samples and subjects

The samples used in the present study were retrieved from the Pediatric Biobank of The First People’s Hospital of Bijie. Cases comprise children who were retrospectively diagnosed with AKI based on pRIFLE (pediatric risk, injury, failure, loss, end stage renal disease) definition, i.e. an estimated creatinine clearance of 50% (as determined from the Schwartz formula) and reduction in urine output below 0.5 ml/kg h for at least 16 h [22]. The baseline creatinine level was assumed to be at 120 ml/min/1.73 m^2^, based on the pRIFLE guideline [22]. Controls were children who had clinical risk factors for AKI and acute lung injury (ALI) but did not eventually develop the condition. (Note: Risk factors for ALI were included because the present work was part of a larger study which also investigates genetic susceptibility to ALI.) Peripheral blood specimens from 1138 cases and 1382 controls were retrieved. These specimens were deposited in the Pediatric Biobank between year 2003 and 2017, and written informed consent was obtained from the parents or guardians of the subjects prior to sample deposition. Before the commencement of the present study, verbal informed consent was re-obtained from either the subjects or their parents/guardians through telephone conversation. We successfully obtained verbal informed consent from all controls and 1129 of the cases. Nonetheless, the Ethics Committee of The First People’s Hospital of Bijie, which reviewed and approved the study protocol, waived the requirements for reconsenting the remaining cases. All subjects were Han Chinese.

### DNA extraction

Extraction of genomic DNA was performed with MagaBio Plus Whole Blood Genomic DNA Purification Kit (Bioer, Hangzhou, China). A volume of 180 µl blood sample was used. Proteinase K and Lysis Buffer were used to lyse the sample, and the DNA released was allowed to bind to MagaBio particles. The bound DNA was captured with a magnet, while the other contaminants were washed twice with the Wash Buffer provided. The purified DNA was eluted in TE buffer (10 mM Tris–Cl, 1 mM EDTA, pH 8.0) and then spectrophotometrically quantified. Only DNA samples with a purity of 1.8–2.0 were included for analysis.

### Polymerase chain reaction

High-Stability PCR Kit (GenScript, Nanjing, China) was used to amplify the DNA regions flanking the polymorphic sites. The primers used for the amplification and their annealing temperature are shown in Supplementary Table S1. Each reaction comprised 1X PCR Buffer, 0.2 mM dNTP, 0.4 mM forward primer, 0.4 mM reverse primer, 150–200 ng DNA template and 2.5 units of Taq polymerase, in a total volume of 20 µl. Thirty (30) cycles of denaturation (94°C, 30 s), annealing (56–61°C, 30 s) and extension (72°C, 30 s) were performed for each PCR reaction. The PCR products were electrophoresed on agarose gel for visualization.

### PCR product purification

PCR Purification Kit (Foregene, Chengdu, China) was used for purification of PCR products. Briefly, 60 µl Buffer BD was first mixed with 15 µl PCR product. The mixture was then transferred into a spin column and full-speed centrifugation was performed. The PCR products were retained on the spin column filter while the smaller molecular contaminants were expelled in the flow-through. Buffer WB1 was then used to wash the PCR products for further removal of the contaminants. The purified PCR product was eluted in 20 µl double-distilled water and quantified by using T60 UV-Vis spectrophotometer (Optoelec, Xi’an, China). Only samples with a purity of 1.8–2.0 were included for further analysis.

### Restriction enzyme digestion and genotype identification

The purified PCR products were incubated with appropriate restriction enzymes as shown in Table 2. Each reaction consisted of 1X restriction enzyme buffer, 10 units of the respective restriction enzymes, and 10 µl of the purified PCR products. Incubation was performed at 37°C for 12–16 h, following which the digested PCR products were electrophoresed on agarose gel. The band sizes were used to identify the genotype (Supplementary Table S2).

**Table 1:** Characteristics of the study subjects

Characteristics	Case	Controls	*P*-value
*N*	1138	1382	–
Age			0.7309
Range	1–16	1–16	
Mean	8.40 ± 4.58	8.46 ± 4.69	
Median	8	8	
Sex			0.2430
Male	596 (52.4%)	756 (54.7%)	
Female	542 (47.6%)	626 (45.3%)	
APACHE II score			<0.0001
Range	15–29	11–26	
Mean	21.8 ± 4.35	18.51 ± 4.65	
Median	22	18	

**Table 2:** Association of inflammation-response gene polymorphisms and risk of acute kidney injury in children

Polymorphism	Case	Control	Odds ratio	*P*-value	Adjusted odds ratio	*P*-value
IL6 rs1800795						
GG	1099 (96.6%)	1349 (97.6%)	Reference		Reference	
GC	38 (3.3%)	32 (2.3%)	1.4576 (95% CI = 0.9047–2.3485)	0.1215	1.3289 (95% CI = 0.8391–2.2182)	0.2189
CC	1 (0.1%)	1 (0.1%)	1.2275 (95% CI = 0.0767–19.6478)	0.8848	1.1083 (95% CI = 0.1381–9.0913)	0.7324
G	2236 (98.24%)	2730 (98.77%)	Reference		Reference	
C	40 (1.76%)	34 (1.23%)	1.4364 (95% CI = 0.9062–2.2767)	0.1233	1.2120 (95% CI = 0.4288–2.8371)	0.4654
IL6 rs1800796						
GG	478 (42.0%)	584 (42.3%)	Reference		Reference	
GC	524 (46.0%)	647 (46.8%)	0.9895 (95% CI = 0.8373–1.1693)	0.9013	0.8417 (95% CI = 0.6237–1.5218)	0.7212
CC	136 (12.0%)	151 (10.9%)	1.1004 (95% CI = 0.8473–1.4291)	0.4731	1.1280 (95% CI = 0.7473–1.8312)	0.8372
G	1480 (65.03%)	1815 (65.67%)	Reference		Reference	
C	796 (34.97%)	949 (34.33%)	1.0286 (95% CI = 0.9155–1.1558)	0.6350	1.0219 (95% CI = 0.8943–1.7832)	0.6037
IL6 rs1800797						
GG	1099 (96.6%)	1348 (97.5%)	Reference		Reference	
GC	38 (3.3%)	33 (2.4%)	1.4124 (95% CI = 0.8800–2.2669)	0.1526	1.3289 (95% CI = 0.8391–2.2182)	0.2189
CC	1 (0.1%)	1 (0.1%)	1.2266 (95% CI = 0.0766–19.6332)	0.8850	1.1083 (95% CI = 0.1381–9.0913)	0.7324
G	2236 (98.24%)	2729 (98.77%)	Reference		Reference	
C	40 (1.76%)	35 (1.23%)	1.3948 (95% CI = 0.8831–2.2031)	0.1536	1.2120 (95% CI = 0.4288–2.8371)	0.4654
IL10 rs1800896						
AA	931 (81.8%)	1104 (79.9%)	Reference		Reference	
AG	187 (16.4%)	255 (18.5%)	0.8696 (95% CI = 0.7064–1.0705)	0.1877	0.5793 (95% CI = 0.8923–1.8932)	0.3204
GG	20 (1.8%)	23 (1.7%)	1.0311 (95% CI = 0.5628–1.8894)	0.9209	1.1901 (95% CI = 0.7281–2.0282)	0.8931
A	2049 (90.03%)	2463 (89.11%)	Reference		Reference	
G	227 (9.97%)	301 (10.89%)	0.9065 (95% CI = 0.7557–1.0875)	0.2906	0.9330 (95% CI = 0.7893–1.1291)	0.3289
IL10 rs3021097						
CC	152 (13.4%)	211 (15.3%)	Reference		Reference	
CT	533 (46.8%)	642 (46.5%)	1.1525 (95% CI = 0.9083–1.4622)	0.2426	1.2398 (95% CI = 0.9083–1.4622)	0.4983
TT	453 (39.8%)	529 (38.3%)	1.1887 (95% CI = 0.9320–1.5162)	0.1638	1.3923 (95% CI = 0.9320–1.5162)	0.2309
C	837 (36.78%)	1064 (38.49%)	Reference		Reference	
T	1439 (63.22%)	1700 (61.51%)	1.0760 (95% CI = 0.9595–1.2067)	0.2100	1.2180 (95% CI = 0.9595–1.2067)	0.2380
NFKB1 rs28362491						
II	552 (48.5%)	464 (33.6%)	Reference		Reference	
ID	477 (41.9%)	662 (47.9%)	0.6057 (95% CI = 0.5107–0.7183)	< 0.0001	0.7482 (95% CI = 0.5834–0.8301)	0.0002
DD	109 (9.6%)	256 (18.5%)	0.3579 (95% CI = 0.2771–0.4623)	< 0.0001	0.4443 (95% CI = 0.3013–0.6732)	<0.0001
I	1581 (69.46%)	1590 (57.53%)	Reference		Reference	
D	695 (30.54%)	1174 (42.47%)	0.5954 (95% CI = 0.5297–0.6691)	< 0.0001	0.6092 (95% CI = 0.4297–0.7329)	0.0001
NFKBIA rs2233406						
CC	832 (73.1%)	942 (68.2%)	Reference		Reference	
CT	289 (25.4%)	390 (28.2%)	0.8390 (95% CI = 0.7019–1.0029)	0.0538	0.9021 (95% CI = 0.3892–0.9936)	0.0018
TT	17 (1.5%)	50 (3.6%)	0.3850 (95% CI = 0.2203–0.6727)	0.0008	0.4335 (95% CI = 0.3280–0.8032)	0.0012
C	1953 (85.81%)	2274 (82.27%)	Reference		Reference	
T	323 (14.19%)	490 (17.73%)	0.7675 (95% CI = 0.6587–0.8944)	0.0007	0.6793 (95% CI = 0.4035–0.8809)	0.0005
NFKBIA rs696						
AA	523 (46.0%)	449 (32.5%)	Reference		Reference	
AG	481 (42.3%)	651 (47.1%)	0.6343 (95% CI = 0.5338–0.7538)	< 0.0001	0.7082 (95% CI = 0.4323–0.8908)	0.0010
GG	134 (11.8%)	282 (20.4%)	0.4079 (95% CI = 0.3205–0.5192)	< 0.0001	0.3892 (95% CI = 0.2114–0.7091)	0.0001
A	1527 (67.09%)	1549 (56.04%)	Reference		Reference	
G	749 (32.91%)	1215 (43.96%)	0.6253 (95% CI = 0.5573–0.7017)	< 0.0001	0.5278 (95% CI = 0.3382–0.8521)	0.0007
IL18 rs1946518						
CC	360 (31.6%)	404 (29.2%)	Reference		Reference	
CA	573 (50.4%)	716 (51.8%)	0.8981 (95% CI = 0.7505–1.0748)	0.2408	0.8728 (95% CI = 0.6893–1.1079)	0.2128
AA	205 (18.0%)	262 (19.0%)	0.8781 (95% CI = 0.6966–1.1068)	0.2709	0.8210 (95% CI = 0.6029–1.2120)	0.2812
C	1293 (56.81%)	1524 (55.14%)	Reference		Reference	
A	983 (43.19%)	1240 (44.86%)	0.9344 (95% CI = 0.8355–1.0449)	0.2340	0.8812 (95% CI = 0.6311–1.1292)	0.2673
IL18 rs187238						
GG	734 (64.5%)	947 (68.5%)	Reference		Reference	
GC	351 (30.8%)	386 (27.9%)	1.1732 (95% CI = 0.9861–1.3958)	0.0715	1.3298 (95% CI = 0.8732–1.5009)	0.1203
CC	53 (4.7%)	49 (3.5%)	1.3955 (95% CI = 0.9352–2.0823)	0.1027	1.7312 (95% CI = 0.9012–2.8392)	0.1931
G	1819 (79.92%)	2280 (82.49%)	Reference		Reference	
C	457 (20.08%)	484 (17.51%)	1.1835 (95% CI = 1.0269–1.3640)	0.0200	1.3214 (95% CI = 1.7712–1.4723)	0.0980
TNF rs1799964						
TT	676 (59.4%)	894 (64.7%)	Reference		Reference	
TC	395 (34.7%)	437 (31.6%)	1.1954 (95% CI = 1.0097–1.4152)	0.0383	1.2100 (95% CI = 1.0128–1.3081)	0.0281
CC	67 (5.9%)	51 (3.7%)	1.7374 (95% CI = 1.1909–2.5347)	0.0039	1.6312 (95% CI = 1.2219–1.9997)	0.0129
T	1747 (76.76%)	2225 (80.50%)	Reference		Reference	
C	529 (23.24%)	539 (19.50%)	1.2736 (95% CI = 1.1119–1.4589)	0.0005	1.3431 (95% CI = 1.1259–1.6127)	0.0117
TNF rs1800629						
GG	909 (79.9%)	1226 (88.7%)	Reference		Reference	
GA	219 (19.2%)	152 (11.0%)	1.9432 (95% CI = 1.5533–2.4311)	<0.0001	1.7073 (95% CI = 1.1328–2.0932)	0.0112
AA	10 (0.9%)	4 (0.3%)	3.3718 (95% CI = 1.0542–10.7852)	0.0045	2.7392 (95% CI = 1.1121–8.8921)	0.0083
G	2037 (89.50%)	2604 (94.21%)	Reference		Reference	
A	239 (10.50%)	160 (5.79%)	1.9095 (95% CI = 1.5503–2.3521)	<0.0001	1.7819 (95% CI = 1.2321–2.2198)	0.0121

### Validation of genotype

Approximately 10% of the purified PCR products were randomly chosen and sequenced using BigDye® Direct Sanger Sequencing Kit (ThermoFisher Scientific, Massachusetts, United States), to validate the genotypes.

### Statistical analysis

Chi-square test and Student *t*-test were used to compare qualitative and quantitative data respectively between cases and controls. A goodness-of-fit Chi-square test was used to measure the deviation of the genotype from the Hardy–Weinberg equilibrium. For the above analyses, a *P*-value of 0.05 was considered statistically significant.

Logistic regression model was used to calculate the odds ratio (OR) for analysis of the association between the polymorphism and AKI risk. The wild-type genotype/allele was used as the reference in the analysis. Three different types of comparison were done, namely (1) heterozygous genotype vs. wild-type genotype, (2) variant genotype vs. wild-type genotype, and (3) variant allele vs. wild-type allele. The ORs were also adjusted for potential confounders (age, sex, APACHE II score) to obtain a more precise estimate of the genetic association. For this analysis, Bonferroni correction was performed to correct for multiple comparison. Thus, a *P*-value of below 0.004 (0.05/12) was considered statistically significant.

All analyses were performed by using SPSS version 22.0 (IBM, Chicago, United States).

## Results

### Subject demographics and clinical features

Samples from 1138 cases and 1382 controls were included in the present study. The demographic and clinical features of the subjects are presented in Table 1. The mean age of cases (8.40 ± 4.58 years old) was slightly lower than that of controls (8.46 ± 4.69 years old), but the difference was not statistically significant (*P* = 0.7309). The age range of both cases and control was 1–16 years old, with a median age of 8 years old. The cases and subjects were also similar in their gender distribution, with 52.4% of the cases and 54.7% of the controls being males (*P* = 0.2430). Besides, cases had a significantly higher APACHE II score (range: 15–29, mean: 21.8 ± 4.35, median: 22) compared with controls (range: 11–26, mean: 18.51 ± 4.65, median: 18) (*P* < 0.0001).

Many other clinical data of the subjects were not available because the samples were retrieved from a biobank and not all clinical data were deposited when the samples were stored.

### Distribution of the polymorphisms

The genotypes of the polymorphisms were successfully determined in all study subjects. Approximately 10% of the samples were validated by sequencing. The concordance rate between RFLP genotyping and DNA sequencing was 100%. No significant deviation from the Hardy–Weinberg equilibrium was observed for all polymorphisms (*P* > 0.05). Interestingly, significant linkage disequilibrium (LD) was observed between the rs1800795 and rs1800796 polymorphisms, especially among the cases, where a total LD was noted.

### Association with risk of AKI

Association between the polymorphisms and risk of AKI in children was considered significant only when the *P*-value was below 0.004 (0.05/12), because Bonferroni correction was performed to correct for multiple comparison. Among the 12 polymorphisms studied, only five (*NFKB1* rs28362491, *NFKBIA* rs2233406, *NFKBIA* rs696, *TNF* rs1799964 and *TNF* rs1800629) showed a statistically significant association when crude logistic regression analysis was performed (Table 2). When adjusted for potential confounders (age, sex, APACHE II score), the statistical significance of the two TNF polymorphisms diminished. On the other hand, *NFKB1* rs28362491, *NFKBIA* rs2233406 and *NFKBIA* rs696 remained to be significantly associated with a reduced risk of AKI. For the *NFKB1* polymorphism, the heterozygous ID genotype showed OR 0.7482 (95% CI = 0.5834–0.8301) (*P* = 0.0002), while the variant DD genotype had an OR 0.4443 (95% CI = 0.3013–0.6732) (*P* < 0.0001). When analyzed at the allelic level, the D allele was found to reduce the risk of AKI with OR 0.6092 (95% CI = 0.4297–0.7329) (*P* = 0.0001).

Similarly, reduced risk was noted for the two *NFKBIA* polymorphisms. The CT genotype of the *NFKBIA* rs2233406 polymorphism showed OR 0.9021 (95% CI = 0.3892–0.9936) (*P* = 0.0018) after adjustment for the confounders, while its TT genotype was associated with reduced AKI risk with OR 0.4335 (95% CI = 0.3280–0.8032) (*P* = 0.0012). At the allele level, the variant T allele showed an OR of 0.6793 (95% CI = 0.4035–0.8809) (*P* = 0.0005). For *NFKBIA* rs696 polymorphism, the AG genotype had an OR 0.7082 (95% CI = 0.4323–0.8908) (*P* = 0.0010) while the GG genotype had OR 0.3892 (95% CI = 0.2114–0.7091) (*P* = 0.0001). The G allele of the polymorphism was also associated with a reduced AKI risk at OR 0.5278 (95% CI = 0.3382–0.8521) (*P* = 0.0007).

## Discussion

Recent evidences suggest that inflammation plays an important role in the pathophysiology of AKI [5,6,23–25]. Polymorphisms in genes involved in the inflammatory process may alter the degree of inflammation in the body, which could influence the risk or susceptibility of an individual to AKI. For this reason, we investigated the association of 12 polymorphisms in six inflammation-related genes with risk of AKI. Among the 12 polymorphisms studied, only five were crudely associated with AKI risk among children in China at *P* < 0.004 (after Bonferroni correction). These include polymorphisms in the NF-κB pathway genes (*NFKB1* and *NFKBIA*), as well as polymorphisms in the *TNF* gene. Interestingly, when the results were adjusted for potential confounders (age, sex, APACHE II score), only polymorphisms in *NFKB1* and *NFKBIA* remained to be significantly associated with AKI risk in Chinese children, whereas the statistical significance of the two *TNF* polymorphisms diminished.

*NFKB1* and *NFKBIA* are two key genes in the NF-κB pathway, which plays an indirect but important role in inflammation. *NFKB1* encodes the NF-κB1 (also named p50) protein, which is the most prominent member of the NF-κB family. NF-κB1 functions as a central coordinator for the activation and regulation of a large array of genes involved in pro- and anti-inflammatory processes, including but not limited to TNF, IL-1β and IL-6 [26]. Thus, NF-κB1 can mediate the inflammation process via various signaling pathways. On the other hand, *NFKBIA* encodes IκBα protein, which is the main inhibitor of NF-κB1. When inflammatory process is not needed, IκBα would bind to NF-κB1 and inactivates the latter. On the contrary, when inflammation is triggered, IκBα would be phosphorylated and degraded, which releases NF-κB1 to the nucleus to regulate the expression of inflammatory genes [27]. Therefore, the two proteins play an indispensible role in the inflammation process.

In this work, we found that the variant allele and genotype of *NFKB1* rs28362491, *NFKBIA* rs2233406 and *NFKBIA* rs696 polymorphisms were significantly associated with a reduced risk of AKI among Chinese children. The *NFKB1* rs28362491 polymorphism is an insertion/deletion variation of the gene, which means that an ATTG sequence is present in individuals carrying the wild type I (insertion) allele and is absent from individuals carrying the D (deletion) allele. This ATTG sequence is important for the binding of nuclear proteins as well as the promoter activity of the gene [28]. Therefore, loss of this ATTG sequence (as observed in D allele carriers) decreases the binding affinity of the promoter sequence and leads to a reduced *NFKB1* promoter activity. As NF-κB1 plays a pivotal role in the inflammatory process, a reduced *NFKB1* promoter activity can result in a low-level inflammation. This explains why children with the *NFKB1* D allele showed a reduced AKI risk in the present study. This is the first study which investigated the association between *NFKB1* rs28362491 polymorphism and AKI risk in children.

On the other hand, *NFKBIA* rs2233406 polymorphism occurs at the promoter region and the variant T allele may disrupt the GATA-2 transcription factor binding, leading to a decreased transcriptional activity of the gene [17]. On the contrary, the variant G allele of the *NFKBIA* rs696 polymorphism can enhance the gene transcription, by reducing the binding affinity of miR-449a microRNA on the gene [29]. Although the two polymorphisms mediate gene transcription in opposite directions, the variant alleles of both polymorphisms were significantly associated with a reduced risk of AKI in children. This illustrates the complexity of the mechanisms by which genetic polymorphisms could affect disease susceptibility. We hypothesize that the rs696 polymorphism plays a more dominant role in influencing the expression of *NFKBIA* gene. This is because the enhancement of *NFKBIA* could result in a stronger inhibition of NF-κB1 protein, which reduces the overall level of inflammation and risk of AKI. Despite this, the interactions between *NFKBIA* rs2233406 and rs696 polymorphisms as well as the exact mechanisms by which they regulate gene expression and AKI susceptibility remain to be elucidated. This is the first study which investigated and found an association of *NFKBIA* rs2233406 and rs696 polymorphisms with AKI risk. However, one previous study (which focused on adult subjects) had also observed a significant association between two other *NFKBIA* polymorphisms (rs1050851 and rs2233417) with AKI risk [30]. These results suggest that NFKBIA (and thus, NF-κB1) could play an important role in mediating AKI risk. This could be an avenue of research in future studies.

In this work, we found that the association of *TNF* rs1799964 and rs1800629 polymorphisms with AKI risk diminished after adjustment for potential confounders. There is only one previous study which investigated the association between rs1799964 polymorphism and AKI risk, and our results concurred with the previous work [31]. On the other hand, *TNF* rs1800629 polymorphism is one of the most frequently studied polymorphisms in AKI [32–37]. Our results were similar to several other previous works, which found no association between the rs1800629 polymorphism and AKI susceptibility [32–34]. However, there were also some previous works which demonstrated an association between the polymorphism and AKI risk (or severity), although conflicting results have been reported [35–37]. These findings demonstrate the complexity of the association, and further studies will be needed for comprehensive elucidation of the role of the polymorphisms in influencing AKI risk.

In this work, we also failed to find an association of the interleukin gene polymorphisms (*IL6* rs1800795, *IL6* rs1800796, *IL6* rs1800797, *IL10* rs1800896, *IL10* rs3021097, *IL18* rs1946518 and *IL18* rs187238) with AKI risk. Among these polymorphisms, *IL6* rs1800795 and *IL10* rs1800896 have been previously investigated with regard to their association with AKI risk [35,36]. Our finding on *IL6* rs1800795 polymorphism agreed to the only previous report on this aspect [36]. However, both previous studies on *IL10* rs1800896 polymorphism showed that the polymorphisms could be associated with AKI risk, which was in contrast with our work [35,36]. The difference between findings in our work and those in previous work could be justified by four explanations. First, association of genetic polymorphisms and disease risk usually varies among different populations [38], and our work is the first one which investigated the association of IL10 rs1800896 polymorphism with AKI risk in Asia. Second, the sample sizes used in previous studies were low, which may cause bias or false positive in the results obtained. Third, the previous studies focused on adult population, while our work focused on pediatric population. Finally, previous studies investigated AKI that has a specific cause, whereas we investigated the injury regardless of its cause (since our aim was to identify the genetic polymorphisms which could predict risk for all kinds of AKI, irrespective of its cause). The lack of significant association for *IL6* rs1800795, *IL6* rs1800796, *IL6* rs1800797, *IL10* rs1800896, *IL10* rs3021097, *IL18* rs1946518 and *IL18* rs187238 in our study suggests that these polymorphisms may play limited roles in susceptibility to AKI.

There are two major limitations in the present study. First, there is a lack of a replication cohort to confirm our study. Without this replication cohort, it could be difficult to rule out the possibility that the association observed was due to chance or other biases in the experimental design. Future studies are needed to replicate our findings in the same population. Second, because the samples were obtained from a biobank, some raw clinical data were not available and no longer retrievable. Thus, the definition of patients is based entirely on the available records. We were not able to check back the raw data to confirm that the diagnosis was correct. Moreover, the baseline creatinine level was set at 120 mL/min/1.73 m^2^ (based on the pRIFLE guideline [22]). This baseline level is controversial [22], and there is a lack of clinical variables to better define the patient population. Thus, there is a possibility that some AKI cases might have been misclassified. Nonetheless, this possibility was small and would not significantly affect the results of our study.

In summary, we have shown that *NFKB1* rs28362491, *NFKBIA* rs2233406 and *NFKBIA* rs696 polymorphisms may be used to predict risk for AKI among Chinese children. Further studies are warranted to replicate our results.

## Abbreviations

AKI: acute kidney injury
ALI: acute lung injury
NF-κB1: nuclear-factor kappa beta 1
